# Editorial: Type 1 interferon in pathologies, autoimmune diseases, and chronic viral infections: understanding the fascinating biologic role of type 1 interferons

**DOI:** 10.3389/fimmu.2023.1239086

**Published:** 2023-07-14

**Authors:** Jean-Philippe Herbeuval

**Affiliations:** Chemistry and Biology, Modeling and Immunology for Therapy (CBMIT), UMR8601, CNRS, Université Paris Cité, Paris, France

**Keywords:** IFN - interferon, autoimmue disease, interferonopathies, self nucleic acid, systemic lupus erythematosus, plasmacytoid dendritic cell (PDC), Toll like 7/8 receptors, interferon regulatory factors

More than 60 years ago a soluble factor which “interferes” with influenza virus infection was discovered named “interferon”. Since then the fascinating complex biology of type I interferons (IFN-I) has captivated researchers from different backgrounds more particularly in virology autoimmunity. Type IFNs are powerful anti-viral proteins capable of inducing hundreds of genes (ISG) essentials for controlling viral infection spread. Paradoxically IFN-I are also implicated in many diseases when produced chronically. Besides the negative impact of IFN-I is well illustrated by a class of disorders termed type 1 interferonopathies composed of rare monogenic diseases complex auto-inflammatory/auto immune diseases such as systemic lupus erythematosus (SLE).

In this 7 papers Research Topic, we covered many aspects of regulation, biological activity in normal or pathologic settings and clinical perspectives.

In his review, Jean-Charles Guery revisits the role of TLR7-driven pDC activation in women during HIV acute and chronic phase (Guery). Type I response is higher in females due to the cell-intrinsic actions of estrogen and X-chromosome complement and is often associated to a better control of viremia and stronger antiviral responses. However recent publication shows that the frequent rs179008 c.32A>T SNP of *TLR7* in women was associated with a lower TLR7 protein synthesis, reduced production of IFN-α by TLR7-activated pDCs, and an unexpectedly lower viral load during primary HIV-1 infection. Jean-Charles Guery reviews the paradoxical role of type I IFN during acute and chronic HIV infection and discuss the recent findings of T allele which seems to be protective.

In the publication of authors demonstrate that treatment of 17β-estradiol increases both interferons-stimulated genes, pro-inflammatory cytokines and chemokines only in PBMC from healthy or systemic lupus erythematous (SLE) female patients. They also show that plasma estradiol levels in female SLE patients, which are higher than in healthy females, positively correlate with the levels of the pro-inflammatory cytokines IL-6, IL-18 and IL-21/23. Taken together, these results highlight the role of estradiol in SLE pathology which may contribute to the higher rate of female suffering from lupus compared to males.

In the study of Yao et al., authors explore the shared gene signature and mechanisms between SLE and Pulmonary Arterial Hypertension using Gene Co-Expression Network Analysis (Yao et al.). They reveal that Type I IFN plays a central role in both diseases. SLE is characterized by impaired cellular organismal processes, persistent inflammation and high activated IFN response. Similarly, in PAH high IFN response forms a complex reciprocal network with the apoptosis, T cell activation and protein ubiquitination, resulting in inflammation and fibrosis. Authors also speculate on novel therapeutic strategies targeting IFN-I pathway that might have potential protective effect on lungs, heart and kidneys in SLE.

In their longitudinal clinical study of SLE patients, Enocsson et al. that galectin-9, which was shown to be correlated to IFN-I and ISG, shows better association with the proinflammatory cytokine TNF than with IFN-α (3). This highlights the incredibly complexity of SLE pathology, with a major role of IFN-I but with also an important inflammatory component. Authors conclude that despite association with IFN-I surrogate biomarkers is therefore not necessarily useful in clinical practice for surveillance of lupus disease activity.

The review of B. Klein and C. Günther focuses on cutaneous DNA damage syndromes with defects in the DNA double-strand repair and nucleotide excision repair (Klein and Günther). They described many possible mechanisms responsible for IFN-I production in this pathology. IFN-I induction represents an anti-tumorigenic mechanism, which may play a pivotal role in inhibiting skin cancer development in the cutaneous DNA damage syndromes. In addition, they also comment recent publications on Ataxia teleangiectasia, Bloom syndrome, Rothmund–Thomson syndrome, Werner syndrome, Huriez syndrome, and Xeroderma pigmentosum. Finally, authors discuss role of IFN-I pathway in cell senescence in which the observed downregulation of IFN-I downregulation may explain the possible failure of immunosurveillance.

Multiple studies described that IFN-I mechanisms contribute to resistance to viral infection and susceptibility to autoimmune disease. In their review article, Sugrue et al. highlight the genetic associations between common single nucleotide polymorphisms (SNPs) in IFN-I pathway that have been associated with both infectious and autoimmune disease (Sugrue et al.). Authors present evidence of a shared genomic IFN-I pathway signature which triggers susceptibility to both conditions using data from multiple SNP association studies. Due to the sex specific effects of SNPs, authors point out the importance of including sex in association studies and think that defining shared transcriptomic and genomic immunological signatures underlying resistance to viral infection and autoimmunity in humans will reveal new therapeutic targets and could improve vaccine strategies.

In addition to classical pathways inducing IFN-I production such as viral RNA or DNA, there are increasing evidences that the loss of mitochondrial integrity permit release of mitochondrial RNA or DNA into the cytosol which can lead to IFN production. In this review article, Lepelley et al. summarize current understanding of how self-nucleic acids released into the cytosol are detected as foreign molecules leading to IFN-I response (Lepelley et al.). Authors focus more particularly on the new findings implicating mitochondrial nucleic acid in type I interferonopathy disease states.

In conclusion, the variety of the publication themes covered in this Research Topic perfectly illustrates the extraordinary diversity of biological role of IFN-I in multiple settings including virology, cancer and autoimmunity. Articles highlight that IFN-I are key proteins in multiple diseases and their shared genomic signature have been associated with both infectious and autoimmune disease. Thus, the identification of genes common to susceptibilities to infections and autoimmune diseases seems to be a way to be better explored to identify new therapeutic targets. Indeed, despite the growing knowledge about IFNs and their signaling pathways, there is still no totally satisfactory therapy for interferonopathies. Although, our understanding of cellular and molecular functions of interferons has tremendously advanced recently, much remains to be learned. urrently, the inhibition of type I IFNs is a captivating and dynamic field of research in therapeutic strategies, showing great promise for various clinical applications (1, 2).

## Author contributions

The author confirms being the sole contributor of this work and has approved it for publication.
